# Effects of Sucrose and Farnesol on Biofilm Formation by *Streptococcus mutans* and *Candida albicans*

**DOI:** 10.3390/microorganisms12081737

**Published:** 2024-08-22

**Authors:** Wit Yee Wint, Mayu Miyanohara, Chika Terada-Ito, Hidenori Yamada, Koufuchi Ryo, Takatoshi Murata

**Affiliations:** 1 Department of Oral Health Science, Tsurumi University School of Dental Medicine, Tsurumi, Yokohama 230-8501, Japan; miyanohara-m@tsurumi-u.ac.jp (M.M.); yamada-h@tsurumi-u.ac.jp (H.Y.); ryo-k@tsurumi-u.ac.jp (K.R.); murata-ta@tsurumi-u.ac.jp (T.M.); 2 Department of Oral Medicine and Stomatology, Tsurumi University School of Dental Medicine, Tsurumi, Yokohama 230-8501, Japan; terada-chika@tsurumi-u.ac.jp

**Keywords:** cell index, biofilm, farnesol, oral pathogens, sucrose

## Abstract

*Candida albicans* (*C. albicans*) and *Streptococcus mutans* (*S. mutans*) are frequently detected in the plaque biofilms of children with early childhood caries. This study investigated the effects of sucrose and farnesol on biofilm formation by the oral pathogens *S. mutans* and *C. albicans*, including their synergistic interactions. Biofilm formation dynamics were monitored using the Cell Index (CI). The CI for *S. mutans* increased in the brain–heart infusion medium, peaking at 10 h; however, the addition of sucrose reduced the CI. For *C. albicans* yeast cells, the CI increased at sucrose concentrations > 0.5%, peaking at 2 h. Mixed cultures of *S. mutans* and *C. albicans* yeast cells showed significantly higher CI values in the presence of sucrose, suggesting a synergistic effect on biofilm formation. Farnesol consistently suppressed biofilm formation by *C. albicans* yeast cells, even in the presence of sucrose, and higher farnesol concentrations resulted in greater inhibition. Regarding *C. albicans* hyphal cells, sucrose did not enhance biofilm formation, whereas farnesol significantly reduced biofilm formation at all concentrations tested. These findings elucidate the complex roles of sucrose and farnesol in biofilm formation by *S. mutans* and *C. albicans* and emphasize the potential of farnesol as an effective oral biofilm inhibitor.

## 1. Introduction

Dental caries, often referred to as tooth decay, is an infectious oral disease that depends heavily on biofilm formation, and early childhood caries (ECC), which affects preschool children, is a growing concern [1,2]. *Streptococcus mutans* (*S. mutans*) is the primary bacterial pathogen responsible for ECC [3,4,5,6]. Similarly, *Candida albicans* (*C. albicans*) is associated with ECC pathogenesis [7,8,9,10]. Moreover, *C. albicans* and *S. mutans* frequently coexist in the oral cavity of children and adolescents [7,11,12,13].

Sucrose, which is fermentable, serves as a substrate for the synthesis of intra- and extracellular polysaccharides (EPS) in dental plaque [14,15]. Frequent exposure to dietary sucrose in the presence of *S. mutans*-derived enzymes such as glucosyltransferases leads to the synthesis of EPS, which are the key building blocks for cariogenic biofilms [16,17]. Studies have revealed an association between sucrose concentration and biofilm formation by *S. mutans* and *C. albicans* [18,19,20,21]. Other studies have investigated the combined ability of *S. mutans* and *C. albicans* to promote biofilm formation [11,22,23,24]. However, the effect of sucrose on the formation of mixed-species biofilms containing *S. mutans* and *C. albicans* remains unknown.

Farnesol, a quorum-sensing molecule produced by *Candida* spp., exhibits antibiofilm activity [25]. Its inhibitory effects on biofilm acid production in *S. mutans* and *C. albicans,* and their number of cultivable cells, have been observed [22,26,27]. Additionally, previous studies have investigated the inhibitory effects of farnesol on mixed-species biofilms containing *C. albicans* and *S. mutans* [22,26]. However, the combined effect of sucrose and farnesol on biofilm formation by *C. albicans* remains unclear. Therefore, it is of particular interest to assess the interactive effects of sucrose and farnesol on biofilm formation in *C. albicans*.

*C. albicans* exhibits two primary morphologies (dimorphic): yeast and hyphae [28]. Hyphal cells adhere more strongly to surfaces and to each other, forming more stable and cohesive biofilms, whereas biofilms formed by yeast cells are less dense, which affects their overall stability [29,30,31,32,33,34]. Interestingly, the effect of sucrose on biofilm formation varies between the two *C. albicans* forms. Yeast cells use sucrose to produce EPS, which aids in the early stages of biofilm formation, whereas hyphal cells rely more on their ability to penetrate and stabilize the biofilm structure through filamentous growth rather than on additional EPS from sucrose [29,35,36]. Most previous studies have focused on the yeast form of *C. albicans*, examining its ability, alone or in combination with sucrose, to induce biofilm formation and the effect of farnesol on this process. However, the role of the hyphal form, both alone and in combination with sucrose, in biofilm formation and the effect of farnesol on biofilm formation by the hyphal form remains unknown. Therefore, this study investigated the individual and combined effects of sucrose and farnesol on biofilm formation by the hyphal form of *C. albicans*.

The Cell Index (CI) indicates the extent and strength of biofilm formation in oral/dental medicine. Monitoring CI variations is helpful for understanding the biofilm-forming capabilities of pathogens. Previous studies have evaluated biofilm formation by oral pathogens, including *S. mutans* and *C. albicans*, by measuring their CI variations using the xCELLigence Real-Time Cell Analyzer (RTCA) AS ONE, Osaka, JAPAN (https://axel.as-1.co.jp/asone/d/3-6815-01/) accessed on 21 August 2024 [23,37,38,39].

This study aimed to assess the effects of sucrose on biofilm formation by single and mixed cultures of *S. mutans* and *C. albicans*. In addition, we aimed to assess the effect of farnesol alone or in combination with sucrose on biofilm formation by the yeast and hyphal forms of *C. albicans*.

## 2. Materials and Methods

### 2.1. Preparation of Culture Media

A Brain–Heart Infusion (BHI) medium (Becton, Dickinson and Company, Sparks, MD, USA), in solid and liquid form, a Sabouraud dextrose agar medium (SDA; Difco, Le Pont de Claix, France), and a Sabouraud dextrose broth liquid medium (SDB; Nissui, Tokyo, Japan) were prepared according to the manufacturer’s instructions.

### 2.2. Source of C. albicans and S. mutans and Culture Conditions

*C. albicans* (ATCC 18804) and *S. mutans* (ATCC 25175; UA159) strains used in this study were sourced from the American Type Culture Collection (ATCC, Manassas, VA, USA). Freeze-dried *C. albicans* was reactivated by culturing it in 10 mL SDB, whereas *S. mutans* was reactivated in 10 mL BHI broth. A few drops of SDB containing reactivated *C. albicans* were streaked onto SDA plates, which were maintained in an incubator at 37 °C for 24–48 h to cultivate new colonies. One single colony was added to 200 µL of the BHI solution and mixed, followed by 100 µL of the *mixed solution* used for the cell index analysis. Similarly, a few drops of BHI broth containing reactivated *S. mutans* were streaked on a BHI solid medium, and one-to-two single colonies obtained were subcultured in 10 mL of a BHI broth liquid medium under anaerobic conditions at 37 °C for 24–48 h to obtain an optical density (OD) of 0.6. From the 10 mL of the BHI solution containing *S. mutans*, 100 µL of the cell solution was used in subsequent experiments with real-time monitoring of CI variations using an xCELLigence RTCA.

### 2.3. Preparation of C. albicans Hyphal Cells

*C. albicans* (ATCC 18804) yeast cells were initially cultured in Tryptic Soy Broth supplemented with 5% dextrose (Becton, Dickinson and Company, NJ, USA) and incubated overnight at 30 °C on a shaker set at 75 rpm to obtain fresh yeast cells. The fresh yeast cells were transferred to an RPMI 1640 medium supplemented with 10% fetal bovine serum and incubated at 37 °C for 48 h to induce the morphological transition from yeast to hyphal form. Following incubation, the hyphal cells were harvested by centrifugation at 2500 rpm for 5 min. The resulting cell pellet was washed twice with sterile PBS to remove any residual RPMI and fetal bovine serum components that could potentially affect the biofilm formation. The washed hyphal cells were resuspended in the sterile BHI solution and used for subsequent experiments.

### 2.4. Effect of Sucrose on CI Variation in C. albicans and S. mutans

BHI solutions containing 0.5%, 1.0%, and 2.0% sucrose were prepared. To assess the impact of sucrose on the CI, the following combinations were introduced into the wells of an E-plate 16 (CA 92121, San Diego, CA, USA):

For *S. mutans*:
200 µL of BHI
200 µL of Phosphate-buffered saline (PBS)
200 µL of BHI with 0.5% sucrose
200 µL of BHI with 1.0% sucrose
100 µL of *S. mutans* and 100 µL of BHI
100 µL of *S. mutans* and 100 µL of BHI with 0.5% sucrose
100 µL of *S. mutans* and 100 µL of BHI with 1.0% sucrose
For *C. albicans* (yeast cells):
200 µL of BHI
200 µL of BHI with 0.5% sucrose
200 µL of BHI with 1.0% sucrose
200 µL of BHI with 2.0% sucrose
100 µL of *C. albicans* and 100 µL of BHI
100 µL of *C. albicans* and 100 µL of BHI with 0.5% sucrose
100 µL of *C. albicans* and 100 µL of BHI with 1.0% sucrose
100 µL of *C. albicans* and 100 µL of BHI with 2.0% sucrose

Two wells were allotted for each combination, and wells containing 200 µL of BHI and 200 µL of PBS were used as controls. The E-plate was inserted into the xCELLigence RTCA and monitored for 21 h. Continuous real-time recording of CIs throughout the experiment allowed for individual plotting on graphs. This experiment was conducted in triplicates for validation.

### 2.5. Effect of Sucrose on CI Variation in Mixed Species of C. albicans and S. mutans

To observe the influence of sucrose on the CIs of the mixed strains of *S. mutans* and *C. albicans* (yeast cells), the following combinations were separately introduced into the wells of an E-plate 16:

200 µL of BHI
200 µL of BHI with 0.5% sucrose
200 µL of BHI with 1.0% sucrose
100 µL of BHI with 100 µL of *S. mutans*
100 µL of BHI with 100 µL of *C. albicans*
100 µL of BHI with 50 µL of *S. mutans* + 50 µL of *C. albicans*
100 µL of BHI with 0.5% sucrose + 50 µL of *S. mutans* + 50 µL of *C. albicans*
100 µL of BHI with 1.0% sucrose + 50 µL of *S. mutans* + 50 µL of *C. albicans*

Two wells were allotted for each combination, and real-time CIs for each treatment were monitored continuously, as described earlier. This experiment was conducted thrice for validation purposes.

### 2.6. Effect of Farnesol on CI Variation in C. albicans (Yeast Cells)

Farnesol (Wako Pure Chemical Corporation, FUJIFILM Wako Pure Chemical Corporation, Osaka, Japan) was diluted in ethanol according to the manufacturer’s instructions. Subsequently, the farnesol solution was diluted in BHI to obtain final concentrations of 1.0, 2.0, 5.0, and 10.0 mM, respectively. To assess the effect of varying farnesol concentrations on the CIs of *C. albicans*, the following combinations were separately introduced into the wells of an E-plate 16:

200 µL of BHI
200 µL of PBS
100 µL of BHI + 100 µL of *C. albicans*
100 µL of BHI + 50 µL of *C. albicans* + 50 µL of 1.0 mM farnesol
100 µL of BHI + 50 µL of *C. albicans* + 50 µL of 2.0 mM farnesol
100 µL of BHI + 50 µL of *C. albicans* + 50 µL of 5.0 mM farnesol
100 µL of BHI + 50 µL of *C. albicans* + 50 µL of 10 mM farnesol

Each combination was added to two wells, and the real-time CIs for each treatment were monitored as previously described. The experiment was conducted thrice to validate the results.

### 2.7. Combined Effect of Sucrose and Farnesol on CI Variation in C. albicans (Yeast Cells)

To assess the combined effect of sucrose and farnesol on the *C. albicans* CI, the following combinations were separately added to the wells of an E-plate 16:

200 µL of BHI
200 µL of BHI + 1.0% sucrose
100 µL of BHI + 100 µL of *C. albicans*
100 µL of BHI + 1.0% sucrose + 100 µL of *C. albicans*
100 µL of BHI + 1.0% sucrose + 50 µL of *C. albicans* + 50 µL of 1.0 mM farnesol
100 µL of BHI + 1.0% sucrose + 50 µL of *C. albicans* + 50 µL of 2.0 mM farnesol
100 µL of BHI + 1.0% sucrose + 50 µL of *C. albicans* + 50 µL of 5.0 mM farnesol
100 µL of BHI + 1.0% sucrose + 50 µL of *C. albicans* + 50 µL of 10 mM farnesol

Two wells were assigned for each combination, and real-time CIs were continuously monitored, as previously described. This experiment was repeated thrice to validate the results.

### 2.8. Individual and Combined Effects of Sucrose and Farnesol on CI Variation in the Hyphal Cells of C. albicans

To assess the effect of sucrose and farnesol on the CI of *C. albicans* hyphal cells, experimental designs were set up similar to those used for the yeast form of *C. albicans*. Similarly, to evaluate the combined effect of sucrose and farnesol on CI *C. albicans* hyphal cells, an experimental design similar to that for the combined effect of sucrose and farnesol on CI variations in *C. albicans* yeast cells was established.

### 2.9. Statistical Analysis

The mean ± standard error CIs of the three replicates after 5 h of incubation were compared using Tukey’s HSD test. Statistical analyses were performed using SPSS version 25 (IBM Corporation, Armonk, NY, USA), and the level of significance was set at *p* < 0.05.

## 3. Results

### 3.1. Effects of Sucrose on Biofilm Formation by S. mutans and C. albicans Yeast Cells

**Figure 1:**
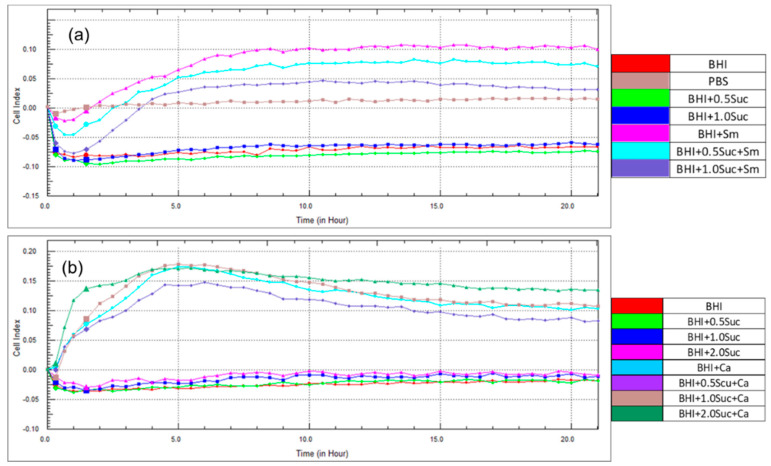
Effect of sucrose on the CIs at different time points. (**a**) Comparison of CIs between BHI, BHI + sucrose (0.5% and 1.0%), and BHI + *S. mutans* (BHI + Sm), BHI + sucrose + *S. mutans* (BHI + Suc + Sm). (**b**) Comparison of CIs between BHI, BHI + sucrose (0.5, 1.0, and 2.0%), and BHI + *C. albicans* yeast cells (BHI + Ca). BHI + Suc + Ca (BHI + Suc + Ca). Abbreviations: CI, Cell index; BHI, brain−heart infusion medium; PBS; phosphate-buffered saline, Suc, sucrose; Sm, *S. mutans*; Ca, *C. albicans*.

**Figure 2:**
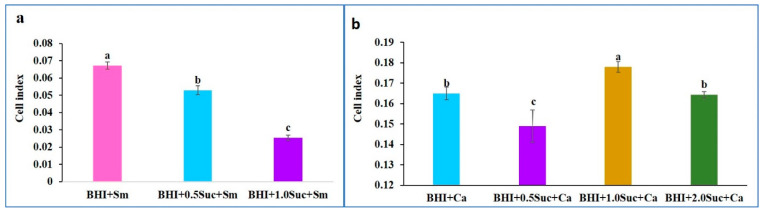
Effect of sucrose on CIs after 5 h of incubation. (**a**) Comparison of CIs between BHI + Sm, BHI + 0.5 Suc + Sm, and BHI + 1.0 Suc + Sm. (**b**) Comparison of CIs between BHI + Ca, BHI + 0.5 Suc + Ca, BHI + 1.0 Suc + Ca, and BHI + 2.0 Suc + Ca. Data represent the means of three replicates, and error bars indicate standard errors. Means with the same letters are not significantly different (Tukey HSD, *p* < 0.05).

Figure 1a,b shows that the CIs were not detectable in the controls (BHI medium or PBS). Furthermore, different concentrations of sucrose in the BHI medium did not enhance the CIs. However, the CI in the BHI medium containing *S. mutans* began to increase after approximately 4 h of incubation, peaked at 10 h, and sustained its peak until 20 h of incubation. Interestingly, the addition of sucrose to the BHI medium containing *S. mutans* significantly reduced the CI. Notably, at 5 h, a greater reduction in the CI was observed at a sucrose concentration of 1.0% than at 0.5% (Figure 1a and Figure 2a).

In contrast to *S. mutans*, in *C. albicans*, the CI was first recorded after 2 h of incubation, peaked at 5 h, but declined thereafter. The addition of 0.5% sucrose to the BHI medium containing *C. albicans* reduced CI values. However, the addition of 1.0 and 2.0% sucrose resulted in higher CIs than those for *C. albicans* alone after 2 h of incubation (Figure 1b). Furthermore, the decline in the CI was faster in *C. albicans* alone compared to those containing sucrose, especially at a concentration of 2.0%. However, after 5 h of incubation, a significantly higher CI than that for *C. albicans* alone was observed only in combination with 1.0% sucrose (Figure 2b).

### 3.2. Effect of Sucrose on Biofilm Formation by Mixed Cultures of S. mutans and C. albicans Yeast Cells

**Figure 3:**
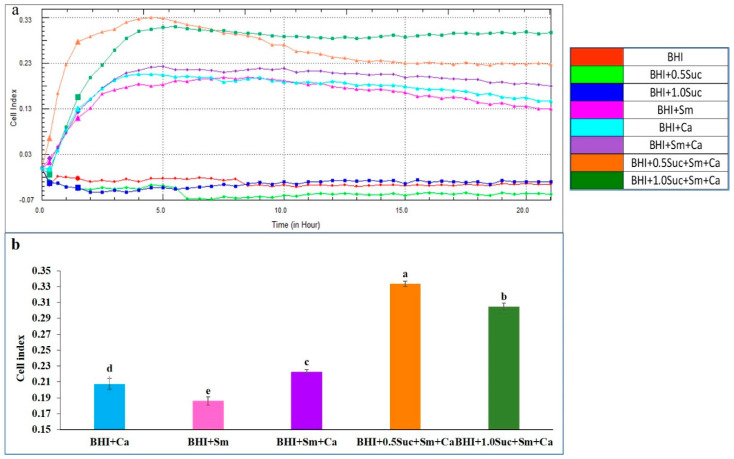
Effect of sucrose on the CIs of mixed cultures of *S. mutans* and *C. albicans*. (**a**) Effect of sucrose on the CIs of mixed cultures of *S. mutans* and *C. albicans* at different time points. (**b**) Effect of sucrose on the CI of the mixed culture of *S. mutans* and *C. albicans* after 5 h of incubation. Data represent the means of three replicates, and error bars indicate standard errors. Means with the same letters are not significantly different (Tukey HSD, *p* < 0.05).

As observed in Figure 1a,b, the CIs were not detected in the BHI medium alone or in combination with sucrose. The CIs of *C. albicans* rapidly peaked after approximately 2 h of incubation and declined thereafter. In contrast, the CIs of *S. mutans* began to increase after 2 h of incubation and gradually increased to reach a peak at 10 h, which was sustained until 20 h of incubation. When *C. albicans* and *S. mutans* were combined, the CI was significantly higher than that of individual species. This was particularly evident after 5 h of incubation (Figure 3a,b), with a slight decline thereafter. Upon the addition of sucrose to the BHI medium containing the mixed culture of *C. albicans* and *S. mutans*, the CI was significantly higher than that of the mixed culture alone and peaked after 5 h of incubation (Figure 3a,b).

### 3.3. Effect of Farnesol Alone or in Combination with Sucrose on Biofilm Formation by C. albicans Yeast Cells

**Figure 4:**
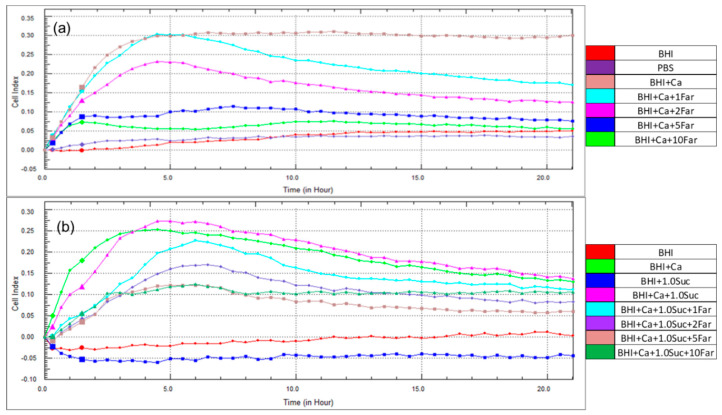
Effect of farnesol on the CIs of *C. albicans* yeast cells at different time points. (**a**) Comparison of CIs between BHI + Ca, and BHI + Ca + Far (1, 2, 5, and 10 mM). (**b**) Comparison of CIs between BHI + Ca, BHI + Ca + 1.0 Suc, and BHI + Ca + 1.0 Suc + Far (1, 2, 5, and 10 mM). Abbreviations: Far, farnesol.

**Figure 5:**
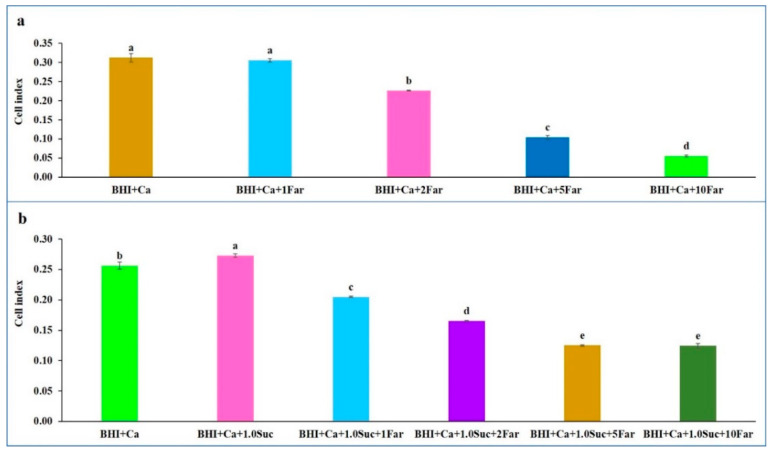
Effect of farnesol CIs of *C. albicans* yeast cells after 5 h of incubation. (**a**) Comparison of CIs between BHI + Ca and BHI + Ca + Far (1, 2, 5, and 10 mM). (**b**) Comparison of CIs between BHI + Ca, BHI + Ca + 1.0 Suc, and BHI + Ca + 1.0 Suc + Far (1, 2, 5, and 10 mM). Data represent the means of three replicates, and error bars indicate standard errors. Means with the same letters are not significantly different (Tukey HSD, *p* < 0.05).

As observed in Figure 1, the CIs were undetectable in the control groups (BHI medium alone or PBS). In the BHI medium containing *C. albicans*, the CI increased after 2 h of incubation, peaked at 5 h, and remained constant throughout the observation period. When a low concentration of farnesol (1 mM) was added to the medium containing *C. albicans* (BHI + *C. albicans* + 1 mM farnesol), the CI remained similar to that of the medium containing *C. albicans* alone after 5 h of incubation. Thereafter, the CI declined over time (Figure 4a). Notably, higher concentrations of farnesol resulted in significantly lower CIs after 5 h of incubation (Figure 5a), confirming its suppressive role in *C. albicans* biofilm formation.

CIs were not detected in the BHI medium alone or in combination with sucrose. However, CIs were observed in the medium containing *C. albicans*, and the addition of sucrose (1.0%) to this medium resulted in higher CIs compared to those without sucrose (Figure 4b), confirming the earlier findings (Figure 1b). After the addition of farnesol (1 mM) to the medium containing *C. albicans* and sucrose (BHI + *C. albicans* + sucrose + farnesol), the CIs were significantly lower than those of BHI + *C. albicans* + sucrose and BHI + *C. albicans*. Significantly lower CIs were observed with increasing concentrations of farnesol up to 5 mM after 5 h of incubation (Figure 5b). Furthermore, sucrose was unable to sustain the biofilm formation when farnesol was added, as the CI for BHI *+ C. albicans* + sucrose + 1 mM farnesol was lower than that for BHI + *C. albicans*.

### 3.4. Individual and Combined Effects of Sucrose and Farnesol on Biofilm Formation by C. albicans Hyphal Cells

**Figure 6:**
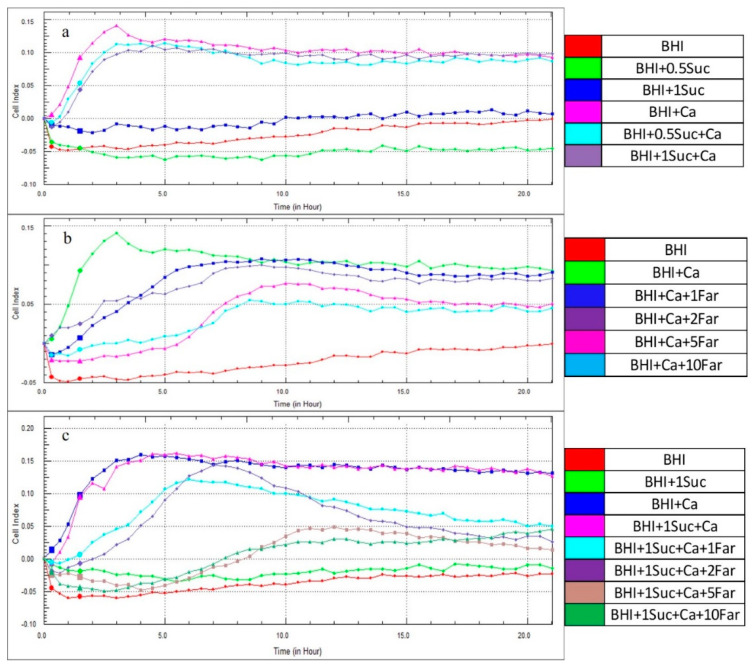
The individual and combined effects of sucrose and farnesol on biofilm formation by *C. albicans* hyphal cells at different time points. (**a**) Comparison of the effects of sucrose on the CIs of *C. albicans* between BHI + Suc (0.5% and 1.0%), and BHI + *C. albicans* (BHI + Ca). (**b**) Comparison of the effect of farnesol on CIs of *C. albicans* between BHI + *C. albicans* (BHI + Ca), and BHI + *C. albicans* with varying concentrations of farnesol (1, 2, 5, and 10 mM). (**c**) Comparison of the combined effects of farnesol and sucrose on CIs of *C. albicans* between BHI + *C. albicans* (BHI + Ca), and BHI + *C. albicans* with sucrose and varying concentrations of farnesol (1, 2, 5, and 10 mM).

**Figure 7:**
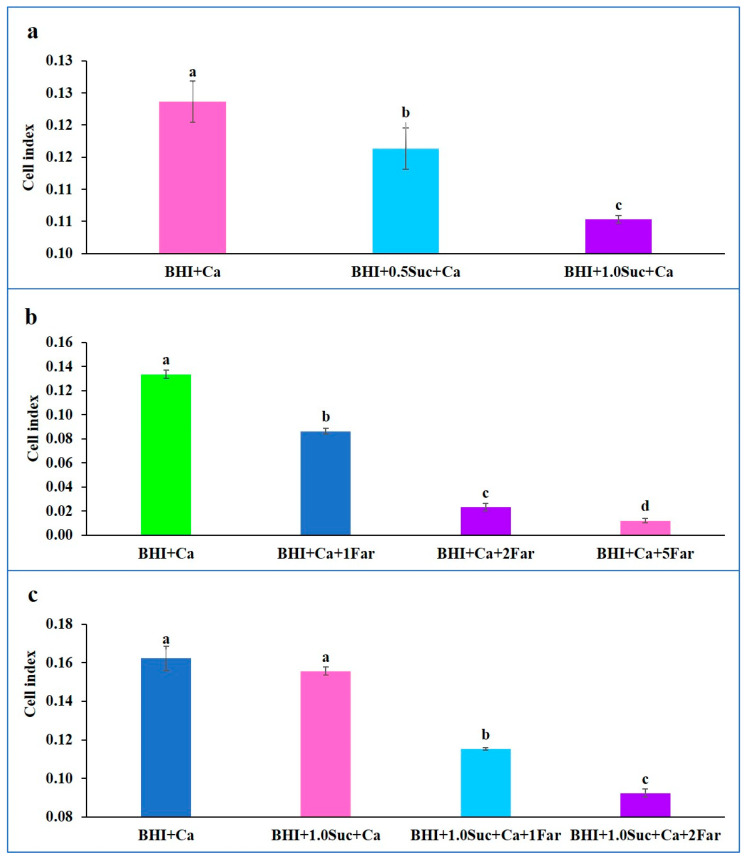
The individual and combined effects of sucrose and farnesol on biofilm formation by *C. albicans* hyphal cells after 5 h of incubation. (**a**) Comparison of the effect of Suc on CIs of *C. albicans* after 5 h of incubation between BHI + Ca, BHI + 0.5% Suc + Ca, and BHI + 1.0% Suc + Ca. (**b**) Comparison of the effect of farnesol on CIs of *C. albicans* after 5 h of incubation between BHI + Ca and BHI + C. albicans with varying concentrations of farnesol (1, 2, and 5 mM). (**c**) Comparison of the combined effect of farnesol and Suc on CIs of *C. albicans* after 5 h of incubation between BHI + Ca, BHI + 1.0 Suc + Ca, BHI + 1.0 Suc + Ca + 1 Far, and BHI + 1.0 Suc + Ca + 2 Far. Data represent the means of three replicates and error bars indicate standard errors. Means with the same letters are not significantly different (Tukey HSD, *p* < 0.05).

CIs for BHI alone or in combination with sucrose (BHI + 0.5 Suc or BHI + 1.0 Suc) did not increase > 0.0, indicating that no biofilm formation occurred in these media. However, the addition of *C. albicans* to the BHI medium (BHI + Ca) resulted in a significant increase in the CI, which peaked after 3 h of incubation and subsequently declined slightly (Figure 6a–c). When sucrose was added to *C. albicans* (BHI + 0.5 Suc + Ca and BHI + 1.0 Suc + Ca), the CIs were significantly lower after 5 h of incubation and slightly lower over the entire period compared to that for BHI + Ca (Figure 6a and Figure 7a). After 5 h of incubation, the CI for BHI + 0.5 Suc + Ca was significantly higher than that for BHI + 1.0 Suc + Ca (Figure 7a). However, after 10 h of incubation, the CI was slightly lower for BHI + 0.5 Suc + Ca than for BHI + 1.0 Suc + Ca, with no significant differences between the two sucrose concentrations.

When farnesol (1, 2, 5, and 10 mM) was added to the BHI medium containing *C. albicans* (BHI + Ca), the CI decreased (Figure 6b). Higher concentrations of farnesol (5 and 10 mM) exhibited a significant inhibitory effect on the biofilm formation by *C. albicans*, as evidenced by the significantly lower CIs compared to those for BHI + Ca after 5 h of incubation (Figure 7b).

Figure 6c shows that the CIs for BHI + sucrose + Ca and BHI + Ca were not significantly different. When farnesol (1 or 2 mM) was added to the medium containing *C. albicans* and sucrose (BHI + Ca + sucrose + farnesol), the CIs peaked after 5 h of incubation, and the values were significantly lower than those for BHI + Ca + sucrose and BHI + Ca (Figure 7c). Increasing the concentration of farnesol to 5 µM resulted in remarkably lower CI values.

## 4. Discussion

*S. mutans* and *C. albicans* are recognized as important oral pathogens that produce biofilms conducive to the development of dental caries [3,4,7,8,9,10,11,40]. Previous studies have reported that sucrose promotes the biofilm-forming capabilities of *S. mutans* and *C. albicans* [18,19,20,21,41]. However, the promotive effect of sucrose on the biofilm formation by *S. mutans* remains unclear in some studies, as its impact on bacterial adhesion and biofilm composition was found to be concentration-dependent [18,42]. Moreover, it remains unknown whether sucrose encourages the joint biofilm formation of *S. mutans* and *C. albicans*. Farnesol has been observed to reduce biofilm formation in these pathogens [22,26,27]. However, the combined effects of sucrose and farnesol on biofilm formation by *C. albicans* remain unexplored. Therefore, we assessed the effect of sucrose on biofilm formation in mixed cultures of *S. mutans* and *C. albicans* yeast cells, as well as the combined effects of sucrose and farnesol on biofilm formation in *C. albicans* yeast cells. Additionally, we examined the individual and combined effects of sucrose and farnesol on biofilm formation by *C. albicans* hyphal cells.

The CI provides real-time data on the changes in cell behavior, including adhesion and proliferation. High CIs indicate increased cell adhesion and proliferation, contributing to biofilm formation. A higher peak CI typically indicates the onset of biofilm maturation, whereas a declining slope after the peak CI value indicates the initiation of the biofilm detachment phase [38,43]. Therefore, in this study, we evaluated the ability of *S. mutans* and C. *albicans* to form biofilms by analyzing their CIs. In this study, the CIs were negative in the controls (BHI medium or PBS), both with and without sucrose. However, noticeable CIs were observed when culturing *S. mutans* and *C. albicans* yeast cells in the BHI medium, confirming the ability of these pathogens to induce biofilm formation, which is consistent with the findings in previous studies [3,4,7,8,9,10,11,40,43]. Interestingly, the presence of sucrose in the medium did not enhance the *S. mutans* CI, and the *S. mutans* CIs tended to be lower at all sucrose concentrations. Previous studies have reported that sucrose, a disaccharide, promotes biofilm formation by oral pathogens as it serves as a substrate for the synthesis of intra- and extracellular polysaccharides (EPS) in dental plaque [18,19,20,21,41]. Similarly, another disaccharide, lactose, significantly stimulates the formation of biofilms by *S. mutans* [44], while the monosaccharide glucose acts as a substrate for intracellular and extracellular polysaccharide synthesis in *S. mutans* and promotes biofilm formation [45,46]. In addition, the polysaccharide glucans promote the accumulation of microorganisms on the tooth surface and contribute to the establishment of the extracellular polysaccharide (EPS) matrix, which provides bulk and structural integrity to dental biofilms [15,20]. Our results contrasted those of previous studies that reported the promotive effect of sucrose on biofilm formation by oral pathogens [18,19,20,21,41]. Cai et al. [18] reported that the effects of sucrose on bacterial adhesion and biofilm composition (dry weight, bacterial count, and EPS content) were concentration-dependent. They observed colony-forming units of *S. mutans* at higher sucrose concentrations. Moreover, Waldman et al. [42] reported that *S. mutans* adhesion initially increased with the increasing sucrose concentration but decreased at higher concentrations. Therefore, although few studies have reported the promoting effect of sucrose on biofilm formation, its role in *S. mutans* biofilm formation remains unclear.

Earlier studies have documented the combined ability of *S. mutans* and *C. albicans* to promote biofilm formation [11,22,23,25]. Our findings align with these results, as observed with the CI values. When considering a single species (either *S. mutans* or *C. albicans*), the CI values were lower compared to the combined *S. mutans* and *C. albicans* biofilms, particularly after 5 h of incubation. This further validates the synergistic effect of these two oral pathogens on biofilm formation, as previously reported [11,22,23,24]. Despite numerous studies reporting the promotive effect of a single species of these pathogens on biofilm formation, the impact of sucrose on biofilm formation by these combined species has not been extensively investigated. Interestingly, although sucrose did not individually promote biofilm formation by *S. mutans*, it notably enhanced biofilm formation when the pathogens were combined. It appeared that the presence of sucrose facilitated the interaction between the two species, favoring an increased biofilm formation when they coexisted. Previous research has indicated the coexistence of *C. albicans* and *S. mutans* in plaque biofilms from children affected by ECC [7,11]. Children and adolescents have a significant consumption of beverages containing sucrose and added sugars, which increase plaque acidity and the potential for plaque formation and bacterial growth in the oral cavity [47,48]. Our study suggested that foods and beverages containing high levels of sucrose should be avoided in children and adolescents to reduce the incidence of dental caries because sucrose significantly increases biofilm formation when *C. albicans* and *S. mutans* coexist.

Oral hygiene, especially interdental hygiene, as well as natural products such as synthesized gold nanoparticles and *Pfaffia paniculata* extract, sodium hexametaphosphate, and fluoride, have been used to test their efficacy against the biofilm formation of *C. albicans* and *S. mutans* [49,50,51,52]. In our study, we observed a suppressive effect of farnesol on *C. albicans* biofilm formation. Notably, the suppression of biofilm formation by farnesol was positively associated with increased concentrations. These results are consistent with those of previous studies [22,25,26,27,53] that reported the ability of farnesol to suppress biofilm formation and reduce the number of cultivable cells of these pathogens. However, the effects of the interaction between farnesol and sucrose on biofilm formation remained unclear. Therefore, we investigated whether farnesol could suppress biofilm formation by pathogens cultured in a sucrose-containing media. Interestingly, farnesol effectively suppressed biofilm formation by *C. albicans* even in the presence of sucrose. Moreover, its suppressive effect on biofilm formation was positively correlated with its concentration.

As described above, sucrose promoted biofilm formation by *C. albicans* yeast cells, and farnesol could inhibit biofilm formation with or without sucrose. In *C. albicans*, hyphal cells are associated with the formation of more stable and cohesive biofilms than yeast cells [29,30,31,32,33,34]. Therefore, we examined the individual and combined effects of sucrose and farnesol on the biofilm formation in *C. albicans* hyphal cells. In contrast to yeast cells, sucrose did not promote biofilm formation by hyphal cells. This discrepancy could be explained by the fact that yeast cells use sucrose to produce EPS, which aids in the early stages of biofilm formation, whereas hyphal cells rely more on their ability to penetrate and stabilize the biofilm structure through filamentous growth rather than on additional EPS from sucrose [29,35,36]. Therefore, the presence of sucrose in the medium did not promote biofilm formation by hyphal cells. However, farnesol strongly inhibited biofilm formation by hyphal cells with or without sucrose, indicating that farnesol has strong anti-biofilm activity against both yeast and hyphal forms of *C. albicans*. Overall, we explored the roles of individual and combined species of *C. albicans* yeast cells and *S. mutans* in biofilm formation. In addition, we revealed how sucrose differently affects biofilm formation by *C. albicans*, depending on its morphology (yeast cells and hyphal cells). Furthermore, we highlighted the suppressive role of farnesol in biofilm formation and examined how farnesol interacts with sucrose in the biofilm formation of *C. albicans* (yeast cells and hyphal cells). The findings presented in this work contribute to a better understanding of the effects of sucrose and farnesol, individually or in combination, on biofilm formation by these oral pathogens.

While the cell index analyzer is valuable for providing real-time and high-throughput data on biofilm formation, it does not directly quantify the bacterial load or assess the biofilm structure, composition, and cell viability. To overcome these limitations, future studies should complement this approach with additional techniques, such as confocal laser scanning microscopy (CLSM), for the detailed visualization of biofilm architecture, live/dead staining for viability assessment, and other methods such as spectrophotometry or flow cytometry for precise bacterial quantification. By integrating these complementary methods, future research can achieve a more comprehensive and accurate assessment of biofilm formations, ultimately leading to a better understanding of the biological processes involved and the potential impact on oral health.

## 5. Conclusions

We have demonstrated the impact of sucrose, farnesol, and their interactions on biofilm formation by the oral pathogens *S. mutans* and *C. albicans* by analyzing CI values. Sucrose exhibited varying effects on the biofilm formation: it reduced CI values for *S. mutans* and *C. albicans* hyphal cells but had a concentration-dependent impact on *C. albicans* yeast cells. Additionally, sucrose promoted biofilm formation in mixed species cultures, highlighting its role in enhancing biofilm formation when *S. mutans* and *C. albicans* coexist. Farnesol emerged as a potent suppressor of biofilm formation by *C. albicans* yeast and hyphal cells, consistently showing lower CI values, even in the presence of sucrose. This inhibitory effect was amplified with increasing farnesol concentrations, underscoring its robust role in biofilm control. The combined effect of farnesol and sucrose further emphasized farnesol’s dominance in inhibiting biofilm formation, outweighing sucrose’s capacity to sustain biofilms when farnesol was introduced. The observed inhibitory effects of farnesol, particularly in conjunction with sucrose, highlight its potential as a significant agent for biofilm control in oral health contexts.

## Data Availability

Data are contained within the article.
